# The North Iowa Medical Society

**Published:** 1873-08-01

**Authors:** J. S. Green

**Affiliations:** Secretary; McGregor, Iowa


### NORTH IOWA MEDICAL SOCIETY.

McGregor, Iowa, June 4th, 1873.

The North Iowa Medical Society held its thirteenth annual meeting in the city council rooms, in the city of McGregor, on Wednesday, June 4th. The President and Vice-President being absent, D. W. Chase, of Elkader, was elected Chairman, *pro tem.*

Minutes of last meeting read and approved.

L. A. Marriam and J. J. Clemmer, of Cresco; H. N. Sill and C. H. Whitney, of Strawberry Point; C. H. Hamilton, of Garnaville; and A. Trueman, of Kendelvill, were duly elected members of the Society. Also, Prof. J. C. Shrader, of the Medical Department of the State University at Iowa City, being present, was elected an honorary member of the Society.

Dr. H. H. Clark, of McGregor, read a lengthy and well written paper upon Obstetrics; gave rules when and how to use forceps. When he was through reading, Prof. Shrader being called upon, reviewed every part of the paper, and gave a practical and interesting address.

Dr. J. T. H. Scott, of Mononee, then read a well written report of his practice for the last year.

Dr. D. W. Chase, of Elkader, gave a minute clinical history and treatment of a lady who had recently and unexpectedly died at that place. The report brought forth some discussion, and it was decided that the disease was puerperal insanity, and that the immediate cause of death was the constant and excessive use of chloroform, administered by officious but well meaning friends, against the advice and objections of the attending physicians.

Dr. J. S. Green, of Postville, reported a case of amputation of the thigh for excessive necrosis of the femur, of nine years' standing the parts had been diseased so long the small arteries had enlarged so that fourteen had to be ligated. Two of the ligatures remained fast for three months.

Dr. S. H. Deake, of Rossville, reported a death from hæmorrhage from the mouth in puerperal convulsions.

Dr. Sill, of Strawberry Point, read a well written essay on Medical Ethics. He also introduced a patient, a young man, with necrosis of the femur, asking for and receiving the opinion of the Society in regard to the treatment of the case.

The Society then proceeded to the election of officers for the ensuing year, which resulted as follows: President, W. C. Earl, of Wawken; Vice-President, S. H. Deake, of Rossville; Secretary and Treasurer, H. H. Clark, of McGregor; Censors, Drs. F. Andrews, J. T. H. Scott, and H. Hamilton.

Delegates to the American Medical Association, J. S. Green and D. W. Chase.

Essayists for the next meeting, Dr. H. H. Clark. To report on Fracture of the Surgical neck of Femur. Drs. Truman, Sill and H. Hamilton. To select their own subjects.

Dr. Green read the following toast: The Medical Profession of the State of Iowa, and the Medical Department of the State University.

Responded to by Prof. J. C. Shrader, of Iowa city. The Prof. spoke in glowing terms of the profession of the State; said that he was well aware that they thought down East that we lived among the wild, uncivilized aboriginies—way behind the times; but he believed the profession in geneial in the State were as well posted, alive, and took as deep an interest in the profession as they did in older States. For proof, look at the well-filled libraries, and the interest that is taken in attending the Medical Societies. He gave the history of the Medical Department of the State University, owned and conducted by the State; showed that it was in a flourishing condition, established on a firm basis, and one of the fixed educational institutions of the land, that every well informed citizen of the State can point to with pride and claim it as his own.

Dr. Scott offered the following resolution which was unanimously adopted:

*Resolved*, That the thanks of this Society be tendered to Prof. Schruder for his attendance, and the interest he has taken in the meeting.

Monona was selected the place to hold the next annual meeting.

Adjourned to meet at Dr. Clark's office at 7 P. M.

Met persuant to adjournment.

Dr. F. Andrews read a paper on the Treatment of Fractures by the Immovable Dressing. Many cases were reported.

J. S. GREEN, M.D., *Secretary.*

---

University Hospital, Philadelphia.—The trustees of this new hospital have nearly six acres of ground, free from all incumbrance, and a building fund, given by the State, of about $200,000. A contribution of $350,000 will also probably be raised. The structure will be 220 feet in length.—*Medical Record.*

